# A theoretical framework for predicting altitude effects on physical employment standard evaluation performance

**DOI:** 10.3389/fphys.2026.1781446

**Published:** 2026-06-22

**Authors:** Matthew Belgiorgio, Martin P. Poirier, Rachel Blacklock

**Affiliations:** Human Performance Research and Development, Personnel Support Programs, Directorate of Programs, Canadian Forces Morale and Welfare Services, Ottawa, ON, Canada

**Keywords:** critical power, hypoxia, intermittent exercise, job task simulation, occupational fitness standards, standard setting, work capacity

## Abstract

Physical employment standard (PES) evaluations assess whether personnel meet an occupation’s minimum physical requirements, supporting operational readiness. Because altitude lowers aerobic capacity, comparability across testing sites at different elevations may require altitude-specific scoring adjustments for aerobic-dominant components. To encourage future efforts to address this, the purpose of this paper is to propose a framework that uses altitude effects observed in existing empirical research to predict altitude effects on PES evaluation performance. No new empirical data are presented; the framework draws on existing altitude–performance literature. We translate altitude effects to PES evaluation performance using four determinants: duration, intensity relative to critical power (CP), work–recovery structure and expected aerobic contribution. The predictions from the framework are expressed as additional completion time at altitude (Δt) and as altitude-specific equivalent cut-scores for a representative aerobic-dominant component with a 360 s sea-level reference, with 7–10% per 1,000 m sensitivity bands above ~1,500 m provided as continuous options. Because aerobic metabolism takes time to dominate an effort, predicted altitude effects scale with task duration: minimal for efforts under 2 min, moderate at 2.5–5 min, and largest at 7.5–10 min. Intermittent work is incorporated using CP and finite work capacity above CP (W′), with W′ reconstitution slowed at altitude so that short bouts with incomplete recovery accumulate fatigue more rapidly than at sea level; full parameter values are reported in the methods. A validation roadmap is outlined that includes within-participant estimation of CP, W′, and recovery kinetics at altitude, translation to PES component demands, and prospective comparison of altitude-specific versus fixed cut-scores under defined exposure timeframes. Such a framework is essential to preserve comparability of PES evaluation outcomes across locations, support defensible decisions based on PES evaluation results, and maintain operational readiness when critical tasks must be performed in hypoxic environments.

## 1 Introduction

Many physically demanding occupations (e.g., law enforcement, fire and rescue, military, paramedic services, and wildland firefighting) require applicants and incumbents to meet physical employment standards (PES). These are typically assessed using evidence-based, job-derived evaluations, including direct task simulations, criterion-related fitness tests, and hybrid protocols combining the two. When developed properly, the PES is reflected in these evaluations with rational and clearly defined performance standards, e.g. cut scores, that are used to determine whether personnel can meet minimum physical requirements linked to safe and effective job performance (Jamnik et al., 2013; Milligan et al., 2016; Petersen et al., 2016; Zumbo, 2016). Members of these occupations may need to perform their duties at elevations above sea level, where reduced atmospheric pressure lowers the partial pressure of inspired oxygen (PIO_2_) and, in turn, arterial oxygen saturation (SaO_2_) via the oxyhemoglobin dissociation curve. Acute compensatory increases in heart rate and ventilation partially preserve oxygen uptake (V̇O_2_) and delivery (Ogawa et al., 2006; Townsend et al., 2016; Park et al., 2022), yet pulmonary and muscular V̇O_2_ kinetics slow during transitions to higher work rates (Weyand et al., 1999; Black, 2011). These constraints narrow aerobic reserve and reduce V̇O_2max_ (Fulco et al., 1998; Wehrlin and Hallén, 2006), with maximal heart rate sometimes declining modestly at moderate altitude, further limiting aerobic capacity (Fulco et al., 1998; Mourot, 2018). In short, aerobic capacity (V̇O_2max_) is reduced in both trained and untrained individuals (Fulco et al., 1998; Wehrlin and Hallén, 2006; Girard et al., 2017; Deb et al., 2018). Above ~1,500 m, V̇O_2max_ and endurance performance typically decrease by ~7–10% per additional 1,000 m on average, with substantial inter-individual variability (Fulco et al., 1998; Wehrlin and Hallén, 2006). Decrements of this magnitude are sufficient to reduce the validity of time-based components of PES evaluations if their standards are determined at sea level (Péronnet et al., 1991; Wehrlin and Hallén, 2006; Beidleman et al., 2016). If minimum physical standards for safe and effective job performance must be met at altitude, sea-level testing may overestimate readiness. Operational contexts demonstrate the stakes: the United States Marine Corps’ Mountain Warfare Training Center (Bridgeport, CA, US) conducts training at ~2,000–3,300 m (Norris et al., 2012); the Indian Army operates in mountainous areas and has reported greater cardiorespiratory strain during fixed-speed load carriage as altitude increases (Chatterjee et al., 2017); and during combat operations in Afghanistan (> 3,000 m), after-action reports describe missions aborted due to altitude illness and severe fatigue (Rodway and Muza, 2011; Nindl et al., 2013).

The importance of understanding how altitude effects translate to PES evaluation performance is not limited to ensuring operational readiness. In contrast to the examples above, a reverse situation is that test-takers that conduct a PES evaluation at altitude without the presupposition of doing their duties at altitude would arguably be unfairly disadvantaged. If sea-level standards/cut-scores are applied without adjustment at altitude, aerobic-dominant PES evaluation components may yield site-dependent bias and increase the risk of false negatives relative to sea-level administration. Across many countries, equality and employment laws prohibit discrimination on protected grounds including age, race, sex, sexual orientation, and disability. Each jurisdiction provides a narrow route for using job-related physical evaluations when a certain level of physical capability is required. In Canada, the applicable route is the bona fide occupational requirement guided by the Meiorin test, which asks whether a standard is rationally connected to the job, adopted in good faith, and reasonably necessary with accommodation to the point of undue hardship (Supreme Court of Canada, 1999). Other countries have similar legal frameworks, such as the United States (United States Congress, 1964; U.S. Equal Employment Opportunity Commission, 1978), the Council of the European Union (2000), and Parliament of Australia (1992). Although the legal tests differ, the common principle is that any physical standard must be job-related, proportionate, supported by evidence, administered fairly, and capable of withstanding legal scrutiny if it is challenged. In practice, this implies that PES evaluation scoring should reflect underlying physical capability rather than site-dependent testing conditions (e.g., altitude), remaining in line with published best-practice guidance on validity and reliability for PES evaluations (Jamnik et al., 2013; Milligan et al., 2016; Petersen et al., 2016; Zumbo, 2016). Best practice would further have test specifications define the environmental ranges within which scores can be interpreted; for altitude, no such specifications currently exist for most occupational PES evaluations, leaving site-by-site decisions to be made without an evidence-based framework. We were able to find only one such example of a real-world altitude scoring adjustment, namely the United States Department of the Air Force Physical Fitness Program, where cut scores for the 1.5-mile run, 2 km walk, and 20 m shuttle run are adjusted for altitude (Department of the Air Force, 2022), although the derivation method is not publicly available. These are also standardized fitness tests rather than job task simulations. Considering the critical need for scientific validity in developing PES evaluations, directly applying single-modality models to job tasks may not be appropriate because task simulations may present distinct physiological demands compared to uni-modal activities such as running or cycling.

Despite these realities, the extent to which altitude affects the validity and comparability of PES evaluations across testing locations has not been systematically addressed. Given the two situations presented above, in both instances there is a need for a model that applies altitude effects across task types and work–rest structures. A pragmatic and novel approach is to translate altitude effects seen in exercise tests to occupational task simulations and then test the predictions against human performance trials at altitude; if prediction error is acceptably small, altitude-adjusted scoring could be justified. Given the lack of literature addressing this issue, the purpose of this paper is to propose a theoretical framework that can be used to, if refined/validated, predict altitude effects on PES evaluation performance to inform valid and defendable altitude-adjusted scoring. While the quantitative examples are literature-anchored and intended to illustrate plausible effects, the article does not present new empirical data. We first consider the altitude effects on different exercise protocols reported in the empirical literature, present the theoretical framework, and present its predictions. Finally, we provide a validation roadmap that outlines recommendations for testing the validity of the framework.

## 2 Altitude effects on performance: an empirical basis for the framework

Throughout this paper, “altitude” refers to elevation above sea level, and “hypoxia” is an umbrella term referring to reduced arterial oxygen availability. The term “hypobaric hypoxia” refers to a hypoxic environment via reduced barometric pressure, which is found either at altitude or simulated in a laboratory. Lastly, “normobaric hypoxia” refers to simulated altitude via sea-level sea-level barometric pressure but reduced oxygen content. Where the source literature uses one modality rather than the other, the modality is specified in the surrounding text; the framework’s primary predictions assume real altitude exposure (hypobaric hypoxia) at moderate elevations (~1,750–3,000 m).

### 2.1 Single-bout performance of aerobic and anaerobic exercise

The focus of this paper is protocol-specific effects that generalize to PES contexts at moderate altitude. Unfortunately, while most research has been focused on using hypoxic conditions as training strategies, less research has investigated the effect of hypoxia on various types of exercise protocols, and this area’s research methodology lacks homogeneity (Girard et al., 2017; Deb et al., 2018). Nevertheless, hypoxic conditions do not appear to negatively affect the anaerobic system. Studies have shown that altitude does not seem to affect muscular strength (Scott et al., 2015b), nor exertion that is heavily dependent on the anaerobic system, such as the Wingate test (Robach et al., 1997; Calbet et al., 2003b). We interpret these single-bout findings as evidence that short, isolated, anaerobic-dominant efforts are often maintained, whereas decrements become greater as aerobic contribution and cumulative oxygen dependence increase with duration or repetition (Girard et al., 2017; Deb et al., 2018). The concepts critical power (CP) and work capacity above CP (W′) provide a useful lens. In basic terms, CP refers to an individual’s maximal steady-state power output, and W’ refers to the total work accumulated above CP until power output can no longer be maintained. Research that estimates CP from time-to-exhaustion trials indicates that W′ is largely preserved in moderate hypoxia even when CP is reduced, with W′ reductions generally reported above ~4,000 m (Morrison et al., 2015; Townsend et al., 2017). During a single short-duration sprint, V̇O_2_ kinetics are slowed in hypoxia (Black, 2011), but anaerobic pathways can partially compensate, so sea-level performance is often maintained if the high-intensity effort is short (Weyand et al., 1999; Ogawa et al., 2005; Goods et al., 2014; Sharma et al., 2019). This pattern aligns with retrospective competition data that showed that across 132,104 track-and-field performances at 794 venues, performance decrements appeared mainly in race distances ≥1,500 m (Hamlin et al., 2015), and at the 1968 Mexico City Olympics (2,400 m), sprint and jump performances were relatively maintained while endurance events were slower (Jokl et al., 1969). One study investigated the effect of hypoxic conditions on performance of a military task. Researchers at the United States Army Research Institute for Environmental Medicine (USARIEM) tested self-paced load carriage during acute altitude exposure, reporting no change at ~2,000 m but slower marching pace at 3,000 m (Coffman et al., 2020).

### 2.2 Intermittent exercise performance with recovery as the moderator

In intermittent exercise, the adequacy of between-bout recovery largely determines the magnitude of hypoxia-related decrements. When aggregated across diverse protocols, effects on intermittent exercise (protocols with longer recovery periods) are small and may be non-significant (Girard et al., 2017; Deb et al., 2018), but short-recovery repeat sprint ability (RSA) subgroups exhibit measurable decrements (Smith and Billaut, 2010; Smith and Billaut, 2012; Billaut and Buchheit, 2013; Bowtell et al., 2014; Goods et al., 2014). Deb et al. (2018) conducted a meta-analysis of 53 studies spanning time trials, time-to-exhaustion, sprints, and intermittent formats; heterogeneity in bout duration, number of sets, and work–rest ratios contributed to the small pooled effects, and analyses restricted to intermittent exercise protocols or bouts <2 min similarly showed no clear effect. Hypoxic environments tend to reduce RSA performance, likely because limited rest increases reliance on aerobic metabolism (Smith and Billaut, 2010; Smith and Billaut, 2012; Billaut and Buchheit, 2013; Bowtell et al., 2014; Goods et al., 2014). By contrast, intermittent protocols with longer recoveries (~3–5 min) show smaller or no decrements (Feriche et al., 2007; Kon et al., 2015), consistent with greater phosphocreatine (PCr) restoration and clearance of accumulated byproducts when oxygen availability and recovery duration are adequate. Accordingly, for PES evaluations composed of multiple components, we infer that the recovery interval between each task becomes a first-order moderator of altitude effects: short recoveries can magnify decrements across later bouts, whereas longer passive/very-light recovery can substantially attenuate between-altitude differences. Taken together, decrements scale with aerobic demand, bout duration, and recovery sufficiency.

### 2.3 Altitude acclimatization

Altitude acclimatization further complicates the administration and interpretation of PES evaluations because time course and magnitude of physiological adaptations vary widely among individuals (Chapman, 2013). Adaptations include increased ventilation, renal bicarbonate excretion with acid–base realignment, plasma volume contraction, and hematological changes that begin within days and can evolve over weeks (Garvican-Lewis et al., 2015; Fulco et al., 1998; Furian et al., 2022). Although V̇O_2max_ may partially recover during the sojourn, aerobic performance typically remains below sea-level values at altitude (Weston et al., 2001; Schuler et al., 2007; Furian et al., 2022). Longer-term performance data remain limited, but aerobic recovery may remain incomplete even after prolonged exposure at higher altitudes (Calbet et al., 2003a). At ~2,210 m, sea-level natives required ~15 months to match altitude natives’ 2.4 km run times at the United States Air Force Academy, coinciding with convergence in hematocrit and hemoglobin (Brothers et al., 2007).

## 3 Framework for predicting the effect of altitude on physical employment standard evaluations

We outline our process of developing a theoretical framework that, if validated, could be used to adjust minimum performance standards in PES evaluations to account for altitude effects. For clarity, we outline the framework within the context of adjusting minimum performance scores originally established at sea level for fair administration at moderate altitude. The framework presented here also assumes short-term acclimatization, while treating acute exposure as a distinct case to be evaluated empirically within the validation roadmap. We synthesize protocol-level performance decrements from the hypoxia literature and translate them to PES evaluation performance decrements using four shared determinants: (i) duration; (ii) intensity relative to critical power (CP); (iii) work–recovery structure; and (iv) expected aerobic contribution (i.e., the proportion of energy supplied oxidatively across a bout or sequence of bouts). The framework is presented first as a duration-based summary and an additional-time-at-altitude mapping (Δt), and then as altitude-specific equivalent minimum standards/cut-scores for a representative aerobic-dominant component.

### 3.1 Prior predictive models

Theoretical and evidence-based models have been developed to predict the effect of altitude on performance. Péronnet et al. (1991) developed a theoretical model of the effect of altitude on running performance and provided predictions of what record-setting sea-level performance times would look like at various altitudes. More recently, researchers at USARIEM conducted time-trial experiments with 95 participants and used the results to develop a quantitative model that predicts changes in task duration as a function of altitude (Beidleman et al., 2016). The latter model has been used to inform operational planning (e.g., acclimatization guidance and workload expectations) and to help mitigate altitude-illness and performance risks (Headquarters, Department of the Army, 2010). A limitation is that these models are based on single bouts of exertion and do not consider the effect of altitude on repeated bouts with rest periods. A further limitation is that they are based on single exercise modalities (i.e., running, cycling) and thus cannot be presumed to be able to be applied directly to PES evaluations. Considering these limitations, we do not build on these models in this paper but rather draw from the primary data in the literature. Altitude-specific scoring adjustments for PES evaluations cannot be considered valid or defendable until a framework, such as the one presented here, is demonstrated to be accurate and able to be applied to task-specific physical evaluations with varying work–rest structures.

### 3.2 Why a CP/W′ work-balance approach

A complementary approach uses the CP framework and the W′-balance model, which describes the depletion and reconstitution of work performed above CP during high-intensity intermittent exercise (Skiba et al., 2012; Skiba et al., 2014a; Skiba et al., 2014b; Skiba et al., 2015). Townsend et al. (2017) reported reduced CP with largely preserved W′ in acute hypoxia and demonstrated the feasibility of W′-balance modeling at ~2,250 m. Applied to tasks with known bout intensities and recovery durations, an altitude-adjusted W′-balance model can generate *a priori* theoretical predictions of evaluation performance at different elevations without immediate field trials. Important limitations remain; the standard equations were validated near ~2,250 m, between-person error for work estimates is relatively large, and parameters were obtained from cycling protocols that may not fully transfer to other modalities or direct task simulations. Accordingly, we treat CP/W′ modeling as a mechanistic translation tool rather than a direct “plug-in” prescription for any single PES component, and we specify validation steps to calibrate model assumptions to PES-relevant modalities and structures.

### 3.3 Mapping dimensions for translation

We translate altitude effects to PES evaluations along four mapping dimensions that determine how much hypoxia will matter for a given task: (i) effort duration (brief ⪅2 min, moderate ~2–5 min, longer ≥5 min); (ii) intensity relative to CP (low, moderate, vigorous, near-maximal); (iii) work–recovery structure (single vs repeated efforts and the length and type of recovery); and (iv) expected aerobic contribution (the proportion of energy supplied from oxidative metabolism across a bout or set of bouts). Because altitude mainly reduces V̇O_2max_ and slows V̇O_2_ kinetics, tasks that are longer, more aerobic, closer to CP, or repeated with insufficient recovery will show larger decrements than brief, isolated, anaerobic efforts (Fulco et al., 1998; Weyand et al., 1999; Wehrlin and Hallén, 2006; Black, 2011; Girard et al., 2017; Deb et al., 2018). In the present illustrations, the duration-based translation is presented separately from the CP and W′ demonstrations. The duration-based curves quantify a global altitude penalty as a function of task duration and do not depend on CP, W′, or recovery kinetics, whereas the CP and W′ figures illustrate mechanisms for intermittent work and recovery.

To preserve comparability of PES outcomes across testing sites, any altitude translation must specify the exposure state to hypoxia under which equivalency is claimed. Unless otherwise stated, the primary predictions and quantitative illustrations presented here assume performance after a short acclimatization window (e.g., ~48–72 h) at the test altitude, with acute exposure (same-day ascent; hours) treated separately in the validation roadmap. With short acclimatization (days) and longer acclimatization (weeks to months), physiological compensation (e.g., ventilatory acclimatization and hematological changes) can partially attenuate decrements in aerobic performance (Garvican-Lewis et al., 2015; Fulco et al., 1998; Calbet et al., 2003a; Brothers et al., 2007; Schuler et al., 2007; Furian et al., 2022); accordingly, the altitude-specific equivalents presented here should be interpreted as reference translations for a defined exposure window, not as universal standards. In practice, programs can operationalize comparability by pairing altitude-specific equivalents with an explicit acclimatization timeframe, and then empirically verifying that pass/fail rates remain stable across altitude sites within that timeframe.

Four exposure states warrant separate consideration when applying altitude-adjusted standards. Acute exposure (same-day ascent; 0–24 h) is dominated by reduced arterial oxygen saturation and slowed V̇O_2_ kinetics, with no benefit from acclimatization-related adaptations; predictions made under this state should use the upper-bound CP decrements and the longer recovery time constant. Short-term acclimatization (~48–72 h) is the state assumed by the primary illustrations presented here and reflects early ventilatory acclimatization with limited hematological change. Partial acclimatization (~7–14 d) reflects substantial ventilatory acclimatization and the onset of plasma volume and hematological adjustment and may support smaller adjustments than the primary illustrations. Prolonged acclimatization (≥ 21 d) reflects more complete adaptation, but performance often remains below sea-level values even after months at altitude (Calbet et al., 2003a; Brothers et al., 2007). Programs adopting altitude-specific equivalents must specify which exposure state applies to a given administration; a single equivalent curve cannot be assumed to apply across all four states without empirical verification.

### 3.4 Parameters for the illustrations

For transparency and replicability, the illustrations use a single, literature-anchored parameterization aligned with moderate altitude (1,750–3,000 m). The framework is a deterministic theoretical model parameterized from the literature, not a regression-fitted statistical model; individual-level variables (e.g., sex, body mass, training history) are therefore not entered as covariates. Instead, the framework operates on test-population-level CP/W′ bounds spanning the range from minimum-standard performance to trained incumbents (sections 3.3, 4.1). Units throughout match the conventions of the underlying CP/W′ literature: power in W, work capacity in kJ, duration in seconds, and decrement as a percentage of sea-level performance. At sea level, we set CP to 250 W and W′ to 20 kJ, representative values from trained adult cycling power–duration (time-to-exhaustion) trials, used only to parameterize the illustrations (Skiba et al., 2012; Skiba et al., 2015; Townsend et al., 2017; La Monica et al., 2018). Because CP and W′ are modality- and protocol-dependent, these absolute values are not mapped 1:1 to job task simulations; the translation relies on relative intensity (to CP) and recovery structure, with a validation approach outlined later (Skiba et al., 2015). We apply CP decrements of ~3% at ~1,750–1,800 m, ~8% at 2,300 m, and ~12% at 3,000 m (Fulco et al., 1998; Wehrlin and Hallén, 2006; Townsend et al., 2017). Reconstitution of W′ during passive or very-light efforts is modeled with a single exponential time constant τ = 300 s at sea level, with altitude-graded scaling specified in the parameterization detail below to reflect plausible slowing in hypoxia (Bogdanis et al., 1996; Haseler et al., 1999; Glaister, 2005; Skiba et al., 2012; Skiba et al., 2014a; Skiba et al., 2014b; Skiba et al., 2015).

**Figure 1:**
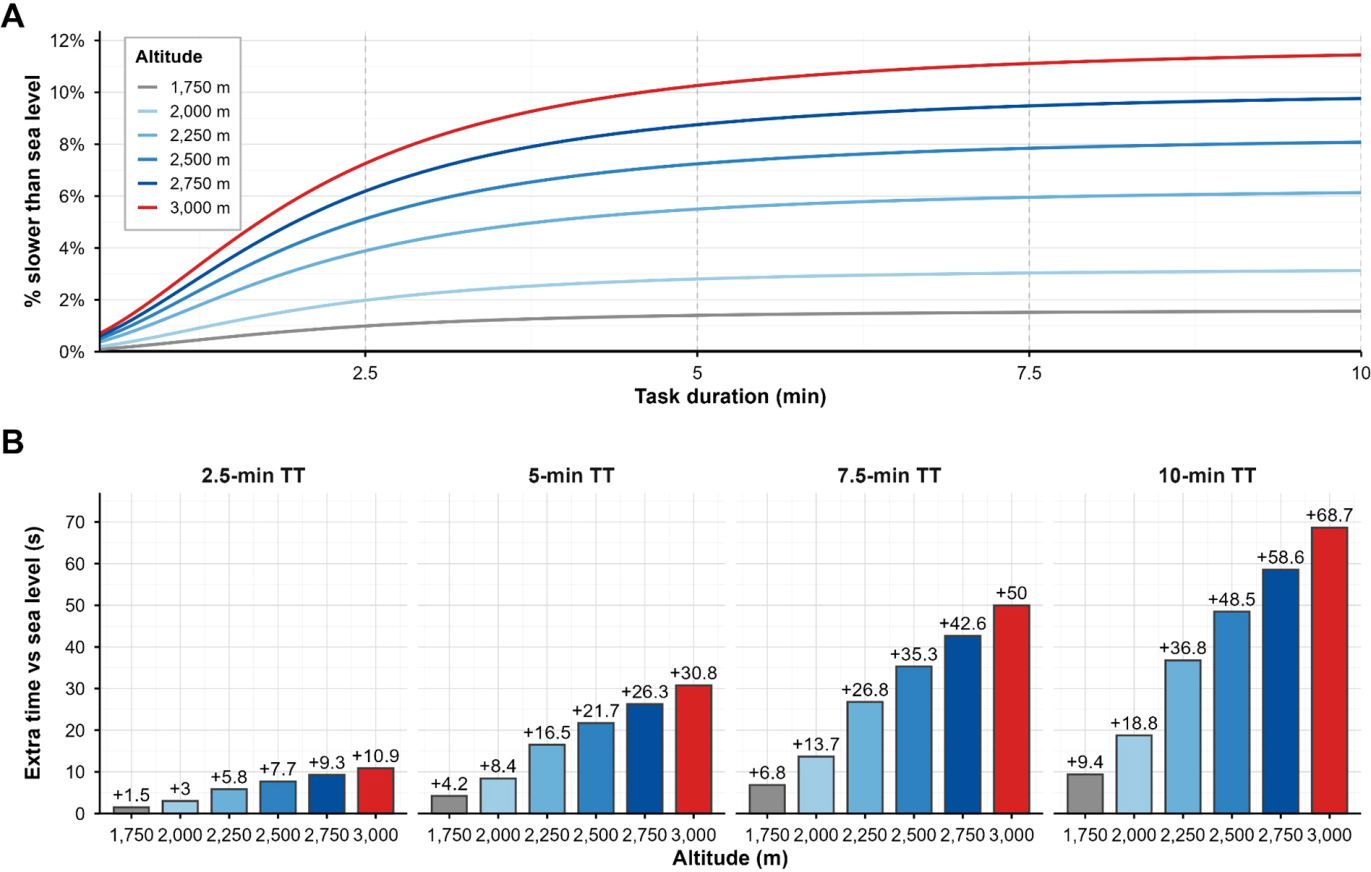
Predicted additional completion time at altitude (Δt) for an aerobic-dominant task across moderate altitude (1,750–3,000 m) under acute to short-term exposure. Panel A shows the nonlinear increase in altitude-related performance decrements with effort duration. Six curves (1,750, 2,000, 2,250, 2,500, 2,750, and 3,000 m) reflect literature-anchored altitude decrements (~7–10% per 1,000 m above 1,500 m) combined with a duration-weighting function in which predicted altitude effects scale with task duration: minimal for efforts under 2 min, moderate at 2.5–5 min, and largest at 7.5–10 min (full parameter values are given in the methods). Panel B converts the same curves into additional time at altitude (Δt) for four representative sea-level task durations (2.5, 5, 7.5, and 10 min). For example, a task completed in 5 min at sea level is predicted to take approximately 30.8 s longer at 3,000 m under the primary assumptions.

**Figure 2:**
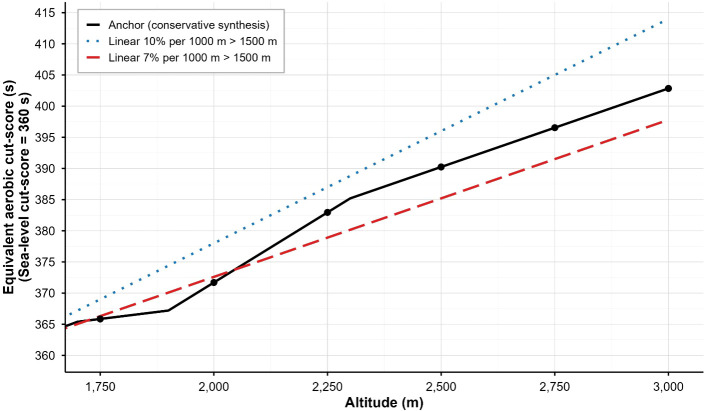
Altitude-specific equivalent cut-scores for an aerobic-dominant component (a task primarily reliant on continuous aerobic effort, here represented by a 360 s sea-level reference time). The black curve shows the equivalent cut-score (in s) at six altitudes between 1,750 m and 3,000 m, illustrating how the time required to demonstrate equivalent performance lengthens with elevation. The dashed line (7% additional time per 1,000 m above ~1,500 m) and the dotted line (10% per 1,000 m) are simpler continuous-function alternatives that programs may use when anchor points are sparse; they bracket plausible adjustment choices and illustrate the implications of more conservative scaling.

We compute additional completion time at altitude (Δt) across representative durations (**Figure 1**) and convert those performance decrements into altitude-specific equivalent cut-scores for a 360 s aerobic-dominant component (**Figure 2**), drawing on established altitude–performance relationships (Péronnet et al., 1991; Fulco et al., 1998; Wehrlin and Hallén, 2006; Beidleman et al., 2016). **Figure 2** also shows linear sensitivity bands of +7% and +10% per 1,000 m above ~1,500 m to bracket plausible choices when data are sparse (Fulco et al., 1998; Wehrlin and Hallén, 2006; Beidleman et al., 2016).

**Figure 3:**
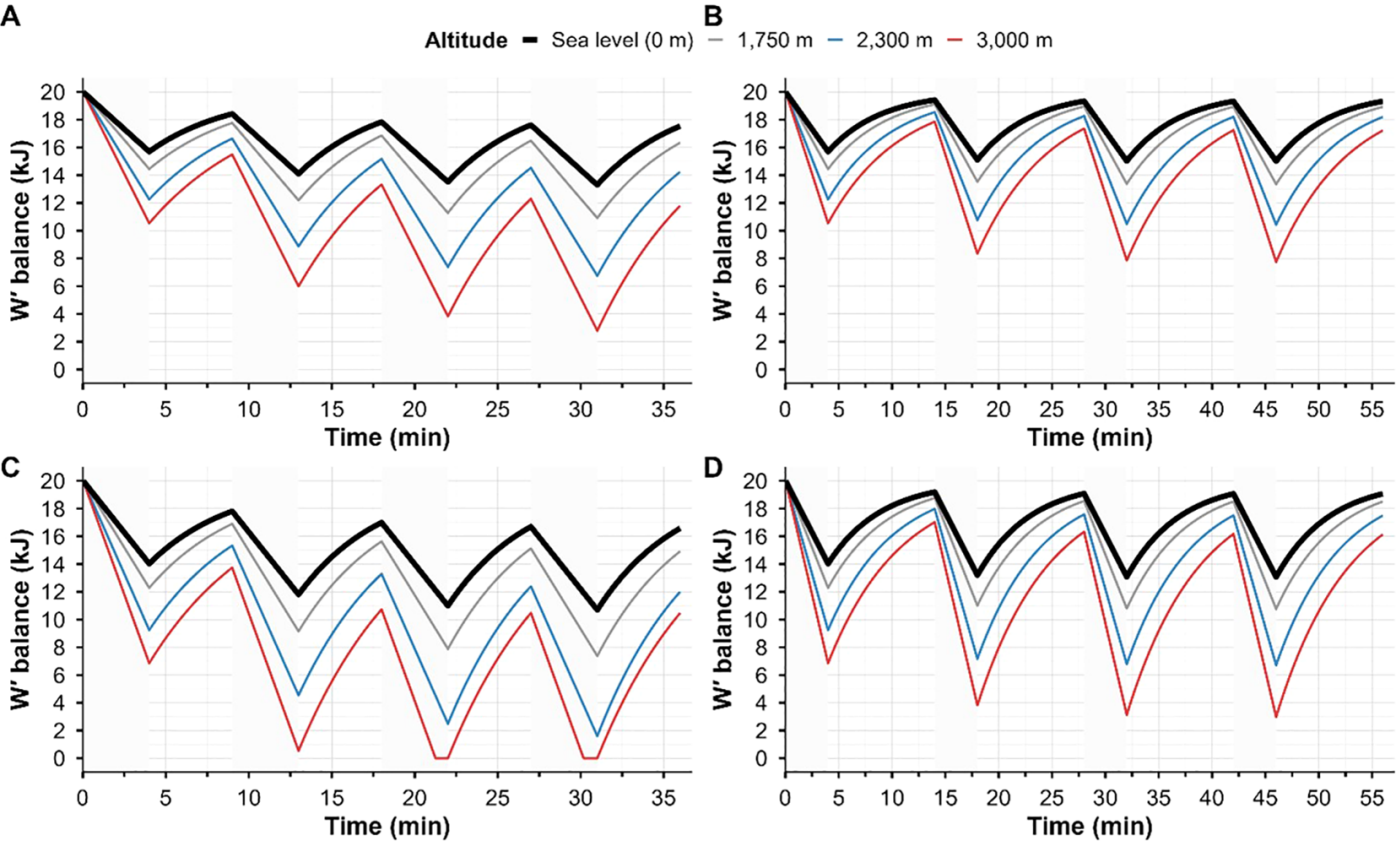
Predicted depletion and recovery of W′ during repeated intermittent work at four altitudes. W′ is the finite work capacity available above critical power (CP, the maximal sustainable power output); when work intensity exceeds CP, W′ is drawn down, and during recovery below CP it is gradually replenished with a time course described by the recovery time constant τ. Predicted W′ balance is shown at sea level and at 1,750 m, 2,300 m, and 3,000 m during four repeated 4-min work bouts performed at a fixed external power equal to 110% of sea-level CP (i.e., 198 W when sea-level CP = 180 W; 275 W when sea-level CP = 250 W), separated by very-light active recovery modeled as 50 W to reflect walking/pacing between bouts. **(A, C)** use 5-min recovery between bouts, whereas panels B and D use 10-min recovery. **(A, B)** represent a lower CP bound (sea-level CP = 180 W, intended to reflect minimally fit/minimum-standard performance); **(C, D)** represent an upper CP bound (sea-level CP = 250 W, intended to reflect a trained incumbent). In the model, altitude progressively reduces CP (up to ~12% at 3,000 m) and slows W′ reconstitution via an increased τ, so that despite identical external work rates the relative intensity above CP increases with altitude, producing greater W′ depletion and slower between-bout recovery. W′ is set to 20 kJ at sea level for all panels; τ is 300 s at sea level and increases with altitude as specified in the model.

For intermittent work (**Figure 3**), outcomes are strongly moderated by recovery timing and type (Haseler et al., 1999; Skiba et al., 2012; Skiba et al., 2015). We make predictions using the CP/W’ construct and W’ balance dynamics, illustrated using lower and upper CP bounds to bracket minimally fit (minimum standard threshold) versus trained incumbents. We simulate four 4-min bouts for two hypothetical people working at 110% of their respective sea-level CP (one ‘lower fitness’ person with a sea-level CP of 180 W, and one ‘trained’ person with a sea-level CP of 250 W) with 105–120% sensitivity bands, separated by very-light active recovery modeled as 50 W for 5 or 10 min. At altitude, a lower CP and a longer τ theoretically decrease performance via increased W’ usage and slower W′ reconstitution; extending recovery from 5 to 10 min reduces between-condition differences, and with 10 min recovery the simulated altitude effect largely disappears, consistent with oxygen-dependent PCr/W′ recovery (Bogdanis et al., 1996; Haseler et al., 1999; Skiba et al., 2014b; Skiba et al., 2015). Together, these illustrations show that altitude effects scale with aerobic dependence, short, isolated tasks (≤2 min) show little change, whereas mid-to-long tasks accrue meaningful Δt, and that recovery duration is the primary modifier in intermittent work (Fulco et al., 1998; Wehrlin and Hallén, 2006; Beidleman et al., 2016).

To facilitate independent replication of the illustrative figures, we describe the modelling environment and the equations used. All computations were performed in R 4.4.0 (R Core Team, 2024) using the tidyverse, scales, and patchwork packages. The duration-based curve in **Figure 1** uses a saturating function, f(t) = D_max · [t²/(t² + t_0_²)], where t is task duration in seconds, t_0_ = 120 s is the half-saturation time, and D_max is the altitude-specific asymptotic decrement. The squared exponent (n = 2) produces an S-shaped curve in which predicted effects are small for very short efforts (where anaerobic capacity dominates), rise rapidly through the 120-s neighborhood (where aerobic and anaerobic contributions are comparable), and approach D_max for efforts beyond ~5 min (where aerobic metabolism dominates). Altitude-specific D_max values (in % decrement of sea-level performance) were anchored at approximately 3% at 1,750 m, 5% at 2,000 m, 7% at 2,250 m, 9% at 2,500 m, 11% at 2,750 m, and 13% at 3,000 m, consistent with the ~7–10% per 1,000 m decrement reported above ~1,500 m (Fulco et al., 1998; Wehrlin and Hallén, 2006). The CP/W′ simulations in **Figure 3** used a 1-second Euler integration of the W′-balance differential equation dW′(t)/dt = −(P(t) − CP) when P(t) > CP, and dW′(t)/dt = +(W′_0_ − W′(t))/τ when P(t) ≤ CP, where P(t) is instantaneous external power, CP is critical power, W′_0_ is the sea-level W′ capacity, and τ is the recovery time constant. CP decrements applied with altitude were ~3% at 1,750 m, ~8% at 2,300 m, and ~12% at 3,000 m (Fulco et al., 1998; Wehrlin and Hallén, 2006; Townsend et al., 2017). These anchors are deliberately conservative relative to Townsend et al., who reported approximately 9%, 13%, and 18% CP decrements at the equivalent altitudes in elite cyclists tested under simulated hypoxia (Townsend et al., 2017). Our smaller decrements reflect (i) the typically lower training status of personnel undergoing PES evaluations, for whom altitude effects on aerobic-dominant performance are generally smaller in relative terms; (ii) the distinction between hypobaric (real altitude) and normobaric (simulated) hypoxia; and (iii) the desirability of avoiding over-adjustment of cut-scores. The sensitivity bands of +7% and +10% per 1,000 m above ~1,500 m shown in **Figure 2** bracket alternative anchors consistent with the higher-end literature. τ was set to 300 s at sea level and scaled by 1.10×, 1.20×, and 1.35× at 1,750 m, 2,300 m, and 3,000 m respectively, as an illustrative assumption representing the oxygen-dependence of PCr and W′ reconstitution under hypoxia (Haseler et al., 1999; Skiba et al., 2012; Skiba et al., 2015). The Skiba et al. (2012) τ formulation (τ_w′ = 546·exp(−0.01·Dcp) + 316) depends on the recovery-power deficit (Dcp) and not directly on altitude; the altitude-graded scaling we apply therefore represents a modelling assumption rather than a calibrated parameter, and direct calibration against altitude-specific recovery data is listed as a priority for future validation work (section 4).

Sensitivity to alternative parameter choices was examined by varying each parameter across plausible ranges and recomputing **Figure 1B**. Halving t_0_ to 60 s steepened the early portion of the duration–Δt curve but produced < ± 15% change in Δt at the 5- and 10-min reference durations. Increasing the sea-level CP from 250 W to 300 W (or decreasing it to 180 W) shifted **Figure 3** W′-balance trajectories but did not alter the qualitative finding that ≥5 min recovery substantially attenuates altitude effects. Increasing the altitude scaling of τ to 1.50× (instead of 1.35×) at 3,000 m increased predicted Δt in the four-bout intermittent simulation by ~20–25% but did not change the direction of the prediction. Across all examined perturbations, the framework’s qualitative predictions, which were minimal effects for short isolated efforts, moderate effects for 2.5–5 min efforts, larger effects for longer efforts, and recovery duration as the dominant moderator in intermittent work, were preserved, but the absolute magnitudes of Δt are conditional on parameter choice and must be calibrated against altitude data before any standard is adjusted.

## 4 Predictions from the theoretical framework

### 4.1 Predicted altitude corrections for PES evaluations

Using the duration mapping in **Figures 1**, **2** gives altitude-specific equivalents for a 360 s aerobic-dominant component across moderate altitude. The anchor curve provides: ~365.9 s at 1,750 m, ~371.7 s at 2,000 m, ~383.0 s at 2,250 m, ~390.2 s at 2,500 m, ~396.5 s at 2,750 m, and ~402.8 s at 3,000 m. These equivalents keep the intended minimum performance requirement constant across testing sites, so “pass/fail” outcomes convey the same meaning regardless of elevation. When programs prefer a single continuous function (e.g., when anchors are sparse), **Figure 2** also shows simple linear sensitivity bands (+7% and +10% per 1,000 m above ~1,500 m) to allow stricter choices without claiming obligation.

Within the moderate-altitude band, we predict small decrements toward the lower end and progressively larger decrements toward the upper end, with minimal effects for brief, isolated, anaerobic-dominant efforts. Extra time allotted to complete a task rises with both altitude and task duration (**Figure 1B**). For example, 5-min efforts add roughly +4.2 s at 1,750 m and +30.8 s at 3,000 m; 10-min efforts add about +9.4 s to +68.7 s across the same range. Using the altitude-specific equivalents (or a uniform altitude scoring curve) prevents testing site-driven shifts in thresholds and point totals when members are evaluated on one score scale.

Finally, **Figure 3** explains why some components need little or no change. In repeated-effort work, altitude decreases performance mainly by lowering CP and slowing W′ reconstitution; extending very-light recovery from 5 min to 10 min markedly reduces between-altitude differences across the sequence. By contrast, decrements should be amplified during repeated efforts when recovery is short or aerobic contribution remains high, consistent with reduced CP and slower W′ reconstitution under hypoxia (Haseler et al., 1999; Weyand et al., 1999; Skiba et al., 2015; Girard et al., 2017). This mechanism aligns with oxygen-dependent PCr/W′ recovery and clarifies why continuous or near-continuous aerobic elements benefit most from altitude-specific equivalents, whereas intermittent elements with enough recovery often remain comparable without further adjustment.

### 4.2 Applying our framework to a real-world example

**Figure 4:**
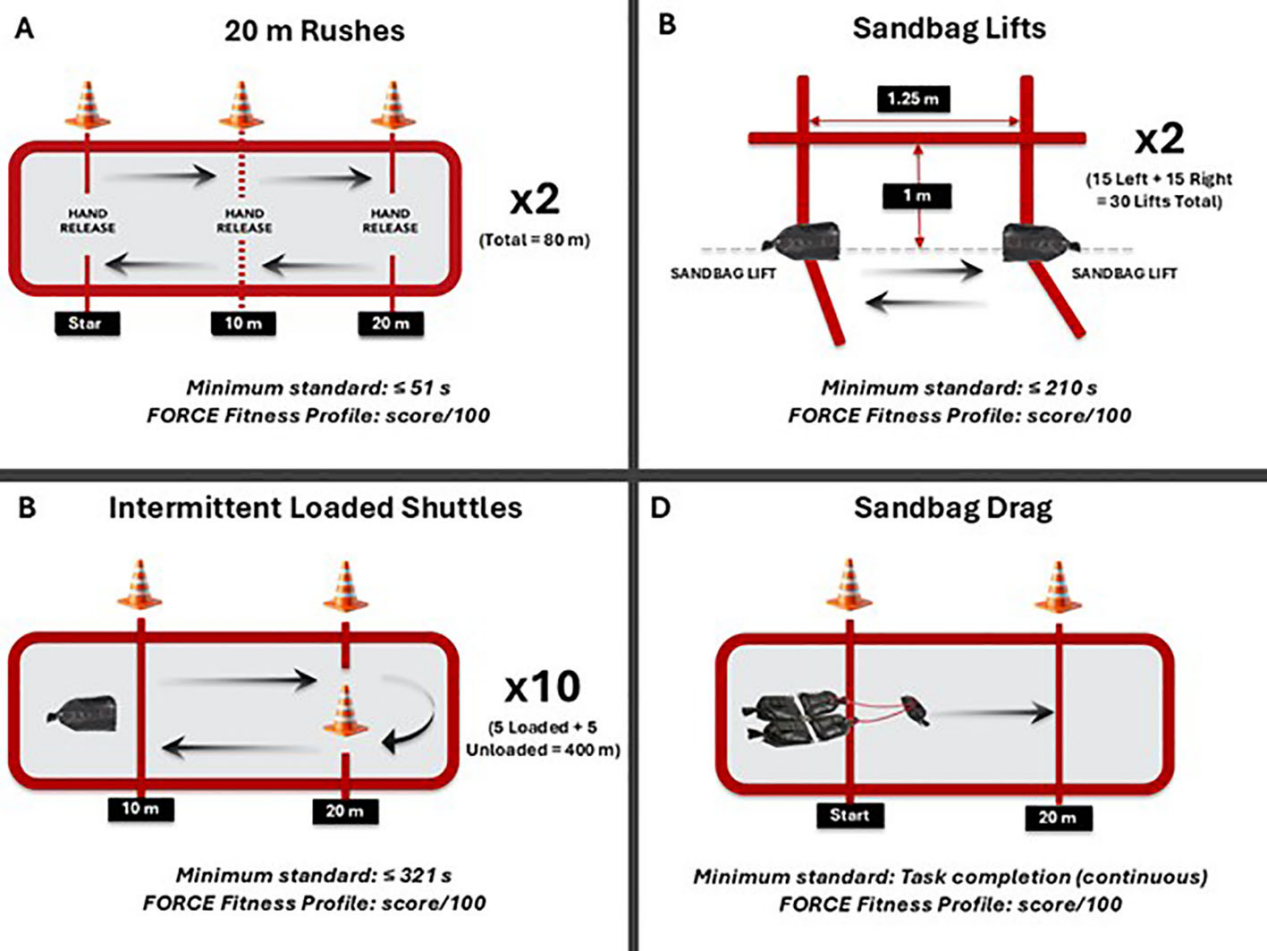
FORCE Evaluation components and administration structure. The FORCE Evaluation comprises four sequential components: **(A)** 20-m Rushes, **(B)** Sandbag Lifts, **(C)** Intermittent Loaded Shuttles, and **(D)** Sandbag Drag. This work–recovery structure mirrors the intermittent scenarios modeled in **Figure 3** and provides the operational basis for expecting smaller altitude effects when recovery is sufficient. Adapted from the FORCE Evaluation Operations Manual (CFMWS, 2023).

The FORCE Evaluation is the Canadian Armed Forces (CAF) annual four-component PES evaluation (20-m Rushes, Sandbag Lift, Intermittent Loaded Shuttles, Sandbag Drag; **Figure 4**), with ≥5 min passive to very-light recovery between components, roughly corresponding to the intermittent recovery structure modeled in **Figure 3**. The evaluation was derived from the Common Military Task Fitness Evaluation (CMTFE) (Canadian Forces Morale and Welfare Services (CFMWS); National Defence, 2006a). Under Universality of Service (National Defence, 2006b), members must meet the minimum standard each year. A subset of personnel (~165) are posted to Peterson Space Force Base, Colorado (~1,887 m above sea level) and are held to the same physical standard (Peterson SFB | Base Overview & Info | MilitaryINSTALLATIONS, 2025). However, the need for a flexible altitude-effect prediction model is highlighted by this evaluation’s nature. The 20 m Rushes involves running and moving in and out of the prone position for 80 m and has a cut-off score of 51 s; the Sandbag Lift involves picking up sandbags and has a cutoff score of 3 min 30 s; the Intermittent Loaded Shuttle involves running and carrying sandbags, and has a cut-off score of 5 min 21 sec; and the Drag involves a 20 m simulated casualty drag with no time limit.

**Figure 5:**
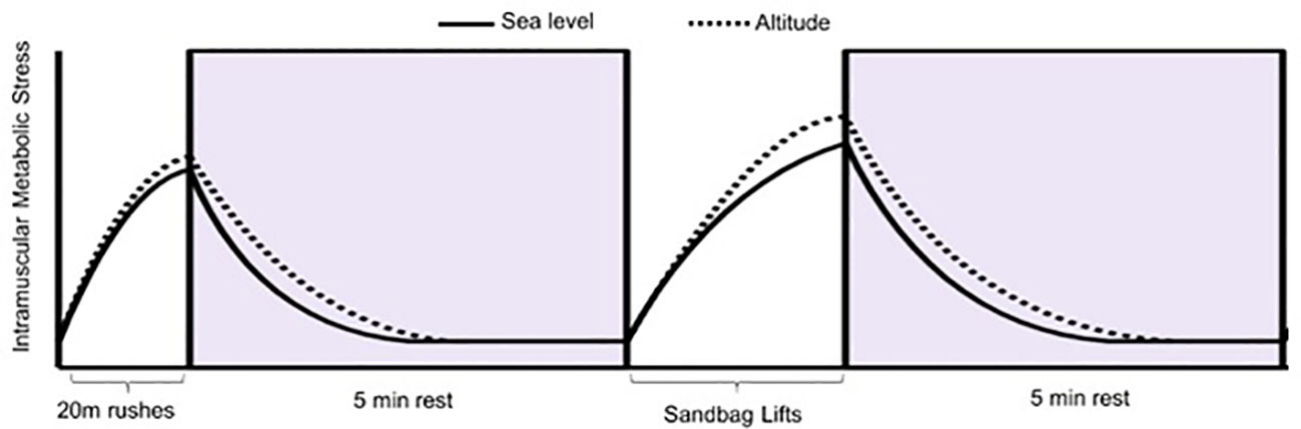
Recovery-dependent mechanism for within-component fatigue at sea level versus ~1,900 m, adapted from Scott et al. (2015a), with permission from Elsevier to the FORCE Evaluation context. The figure illustrates how phosphocreatine (PCr) and W′, the muscular energy stores drawn upon during high-intensity work, recover between components in an oxygen-dependent manner. At altitude, lower oxygen availability slows their reconstitution, and greater per-bout oxygen deficit also leads to more accumulation of metabolic by-products (e.g., hydrogen ions, inorganic phosphate) that contribute to fatigue. With ≥5 min of very-light or passive recovery between components, these altitude-related effects are largely offset; with shorter recovery, decrements may emerge in later components. This mechanism supports the prediction of minimal altitude effects for the FORCE Evaluation configuration at ~1,900 m.

Under typical administration (≥5 min very-light recovery between components), the present framework predicts that minimum standards are unlikely to require adjustment at this elevation after a defined short acclimatization window. This is in line with what we would hypothesize when considering the relevant literature. We reviewed eight intermittent/interval studies at real or simulated altitudes ≥1,750 m, and their results are summarized in **Table 1**. We included only studies investigating altitude effects on performance, excluding research on altitude as a training strategy. To align with the FORCE Evaluation work–recovery structure, we included only studies using intermittent exercise protocols involving at least two repeated bouts or sets each lasting no more than 5 minutes, interspersed with at least 150 s of passive or active rest. Four reported no decrements with ~3–5 min recovery or otherwise sufficient rest (Robach et al., 1997; Feriche et al., 2007; Kon et al., 2015; Karayigit et al., 2022), whereas the four with shorter recovery times showed small decrements concentrated in later bouts (Brosnan et al., 2000; Goods et al., 2014; Morrison et al., 2015; Zinner et al., 2015). A primary explanation is oxygen-dependent recovery: PCr resynthesis tracks VO_2_ kinetics and oxygen availability, both lower in hypoxia; decrements diminish as between-bout recovery approaches ~3–5 min (Bogdanis et al., 1996; Haseler et al., 1999). A complementary view is greater intramuscular metabolic stress across repeated efforts in hypoxia, which accumulates and can impair performance in later bouts (Glaister, 2005; Scott et al., 2015b). **Figure 5** adapts that threshold-style schematic to the FORCE Evaluation context and aligns it with the CP/W′ mechanism in **Figure 3** (Scott et al., 2015a). To be clear, the final decision to make/forego altitude adjustments to this evaluation should not be made before either the validation of the prediction model or direct investigation using methods described above.

**Table 1:** Overview of research on the effect of altitude on intermittent exercise performance.

Reference	Participants	Altitude/intervention	Testing protocol	Measured outcomes	Effect of altitude
Robach et al., 1997	Moderately trained males (n=5) and females (n=2)	5 d at 4,350 m (real)	2 x 20-s Wingate, separated by 5 min of rest	Power output (W/kg)	No effect
Brosnan et al., 2000	Elite female cyclists (n=8)	~2,100 m (simulated) FIO_2_: 17.42%	3 sets of 6 x 15-s sprints with 3 min passive rest	Power output (W)	↓ 5.0 ± 4.0%
Feriche et al., 2007	Male physical education students (n=8)	690 m (baseline) vs. 2,320 m (real)	5 x 400 m treadmill intervals with 1-, 2- or 5-min passive rest	Distance run (m, # of sprints)	No effect
Goods et al., 2014	Soccer players (n=10)	2,000, 3,000, & 4,000 m (simulated)	3 sets of 9 x 4-s sprints with 3 min passive rest	Power output (W)	↓ 6.2% at 2,000 m
Kon et al., 2015	Endurance trained male cyclists (n=7)	2,000 m (simulated)	4 x 30-sec cycle sprints, separated by 4 min rest	Peak power output, Mean power output (W)	No effect
Morrison et al., 2015	Amateur team athletes (n=10)	~3,200 m (simulated with FIO_2_ 14%)	4 sets of 4 x 4-s sprints with 26-s rest (2:26 min rest between sets)	Speed (m/s) Distance run (m)	↓ 1% in average speed in sets 3 and 4
Zinner et al., 2015	Endurance trained male athletes (n=10)	~1,800 m (simulated with FIO_2_ 16.5%)	3 x 3-min on a cross-country ski ergometer, separated by 3-min rest	Power output (W)	Set 1: ≠ Set 2: ↓ 4% Set 3: ↓ 6.5%
Karayigit et al., 2022	Male (n=13) and female (n=13) team-sport athletes	2,500 m (simulated)	6 x 15-s sprints with 2-min active recovery	Peak power (W) Mean power (W)	No effect

FIO_2_, Fraction of inspired oxygen; m, metres; min, minutes; s, seconds; m/s, metres per second; W, watts; ↓, decrease; ↑, increase; statistical significance set at p ≤ 0.05 for all analyses. Studies were included if they (i) investigated altitude effects on performance rather than altitude as a training intervention and (ii) used intermittent exercise protocols involving at least two repeated bouts or sets, each lasting no more than 5 min, separated by at least 150 s of passive or active recovery. These inclusion criteria were chosen to align with the work-recovery structure of multi-component PES evaluations such as the FORCE Evaluation. Studies using single-bout time-trial or graded exercise designs are not included because their work-recovery structures do not match the operational PES context.

## 5 Validation roadmap

This section turns the framework into concrete, testable predictions and a plan to check them across the full moderate-altitude range of 1,750–3,000 m in 250–500 m steps (both acute exposure and after a short acclimatization window where feasible).

1. Mechanism check (CP, W′, recovery kinetics): Within-participant tests across altitudes should show that CP decreases progressively with elevation, W′ remains largely preserved within moderate altitude, and the effective recovery constant (τ) during passive/very-light recovery lengthens with elevation. These patterns should be sufficient to reproduce the direction and shape of the Δt curves, and the narrowing W′-margin seen in the figures.
2. Task translation to PES components: For each component, quantify intensity relative to CP and the between-task recovery used in PES administration. Predictions are that components that sit near CP and/or repeated efforts with short recovery will exhibit larger Δt, while brief (≤2 min), isolated efforts with ≥5 min very-light recovery will show minimal Δt. Empirical Δt at altitude increments should align with the duration-based mapping (**Figure 1**) and produce the altitude-specific equivalents (**Figure 2**) when applied to a 360-s aerobic-dominant reference.
3. Comparability tests for standards: Evaluate hypothetical altitude-specific equivalent cut-scores (**Figure 2**) to compare results gathered across altitudes and confirm that pass/fail rates would remain stable across sites. Using a fixed sea-level standard at altitude should create predictable site-dependent shifts (more test scores near, on or below the cut-score for aerobic-dominant components), whereas the altitude-specific equivalents should remove those shifts.
4. Falsification/decision rules: Pre-define simple falsification checks: (a) if CP does not decline with elevation and τ does not slow, the mechanism is undermined; (b) if altitude-specific equivalents fail to stabilize pass/fail rates across testing sites, the scoring translation should not be adopted; (c) if extending recovery from 5 → 10 min doesn’t compress Δt and widen W′-margin, the intermittent prediction is not supported.

To test these predictions, future work should prioritize prospective, within-participant studies across multiple elevations within the moderate-altitude band, with standardized sea-level baselines immediately pre-departure. Key elements include collecting CP, W′, and τ alongside task-simulation performance; standardizing warm-up, task order, and ≥5-min inter-component recovery; logging barometric pressure and other environmental conditions; and indexing physiological strain (SpO_2_, heart rate). Samples should be age- and sex-stratified to preserve the external validity of the findings. Altitude-adjusted work–balance parameters need to be calibrated to task-simulation data beyond cycling, with acceptable prediction error thresholds defined *a priori* before any adoption of adjusted scoring (Skiba et al., 2012; Skiba et al., 2014b). Recovery-threshold experiments should manipulate inter-bout and inter-component recovery (e.g., ~3–6 min) to identify durations that restore comparability for intermittent elements while verifying that continuous/aerobic tasks remain more altitude-sensitive (Haseler et al., 1999; Glaister, 2005). Acclimatization trials should compare short, staged timeframes (e.g., ~48–72 h, ~7–14 d, ~21–28 d) and report sex-inclusive outcomes using standardized exposure metrics (barometric pressure and/or inspired O_2_ fraction), building on military acclimatization research (Beidleman et al., 2008; Fulco et al., 2011). Finally, implementation studies should test whether altitude-specific equivalents preserve pass/fail performance distributions across sites and should audit false negatives/positives and policy defensibility in real programs.

### 5.1 Acceptable prediction error and tiered implementation

As mentioned in the Introduction, we envision two use cases where this framework, once validated/refined, could be implemented. The first use case is that focused on in this paper—the instance where individuals are being physically evaluated at altitude for an occupation without the pretense of having to do that occupation at altitude (i.e., the employer would make the evaluation “easier” to account for the hypoxic environment). These altitude-adjusted scoring rules should not be adopted operationally until validation studies demonstrate an absolute prediction error within ±5% of completion time at the relevant moderate-altitude band, and a difference in pass/fail rates of no more than ±5 percentage points relative to sea-level administration after applying the altitude-specific equivalents. Altitude-specific cut-scores would be implemented as the official standard for testing administered above ~1,500 m only after the ±5% thresholds above have been met for that PES evaluation, with prospective re-validation on a defined schedule (e.g., every 5 years or whenever job demands change).

The second use case involves the contrasting situation where it is realized that an occupation with a PES evaluation administered at sea level may involve real-world scenarios where the occupation needs to be performed at altitude. For example, cut-scores for a time-trial PES evaluation at sea level would be *shortened* to ensure the person could compensate for a hypoxic environment and perform their duties at altitude. Until those thresholds mentioned above are met, programs may consider a tiered implementation pathway. Tier 1 (Readiness Monitoring) uses the framework’s predictions internally for non-punitive readiness monitoring without affecting any pass/fail decision, allowing programs to accumulate within-organization data without legal exposure. Tier 2 (Informational Equivalents) reports both the sea-level and the altitude-specific equivalent score on the test sheet but applies only the sea-level cut-score for employment decisions; this allows incumbents and applicants to see the predicted altitude effect and gives the program a parallel data stream against which to validate the framework. Tier 3 (Adopted Equivalents) involves adoption of a new cut-score that takes into account the requirement of performing the job at altitude. However, we emphasize that such a scenario would mean legally exposing the employer for the benefit of employee readiness for possible altitude scenarios. It would be advisable that punitive altitude-adjusted scoring should be done in a conservative manner in this scenario.

## 6 Limitations

This work synthesizes heterogeneous studies spanning exercise modalities (e.g., running, cycling, skiing), exposure types (normobaric vs. hypobaric hypoxia), and protocol structures (continuous, repeated-sprint and intermittent formats). Studies vary in the mode of exercise (running, cycling, Nordic skiing), the type of exercise protocol (e.g. time-to-exhaustion, time trial, etc.), participant characteristics (sex, V̇O2max/V̇O2 kinetics), the dose of hypoxia (real or simulated), and the hypoxic condition (normobaric hypoxia, hypobaric hypoxia, or real altitude). The use of varying types of hypoxic conditions makes drawing conclusions difficult since there is evidence that hypobaric and normobaric hypoxia can elicit different physiological responses during exercise (Treml et al., 2020). Overall, this heterogeneity constrains data pooling and invites protocol-specific bias (Deb et al., 2018; Treml et al., 2020). For PES translation, this heterogeneity matters because “altitude effects” are not a single constant. The magnitude depends on duration, intensity domain, and recovery structure, which are exactly the features that PES evaluations combine in different ways.

Our quantitative illustrations use a single parameter set (CP = 250 W; W′ = 20 kJ; τ = 300 s with a modest slowing at altitude) and “moderate-altitude” anchors within ~1,750–3,000 m derived largely from endurance/cycling evidence on V̇O_2_max decrements and intensity-domain shifts; transfer to mixed-modality task simulations is inferential and not yet validated (Fulco et al., 1998; Wehrlin and Hallén, 2006; Skiba et al., 2012). Inter-individual responses to hypoxia are variable and incompletely explained, and women remain under-represented in many hypoxia–exercise datasets, limiting sex-specific inference (Fulco et al., 1998; Chapman, 2013). However, a recent review did note evidence for sex differences in the physiological responses to hypoxia (Raberin et al., 2024). In our context, this may mean different altitude adjustments for male and female test-takers. Finally, operational data used to check the framework can be affected by non-standardized testing.

Beyond the sex-differences caveat noted above, recent reviews indicate that women may exhibit distinct ventilatory, haemodynamic, and metabolic responses to hypoxia (Raberin et al., 2024; Burtscher et al., 2024), and these differences are sufficient in magnitude that future validation studies of altitude-adjusted PES standards must be powered to detect sex-specific effects and report outcomes stratified by sex. Several additional moderators warrant explicit acknowledgement as boundary conditions for the framework. Age affects both the magnitude of altitude-related decrement and acclimatization kinetics; menstrual-cycle phase has been associated with shifts in ventilatory response and core temperature regulation that may interact with hypoxia; prior altitude exposure (within the preceding ~3 months) can attenuate decrements via residual ventilatory and haematological adjustments; and acute hydration and glycogen status influence aerobic performance independently of altitude. The framework presented here assumes test-takers are properly fuelled and hydrated and have no recent altitude exposure. Validation studies are encouraged to record and report these moderators, and operational programs adopting Tier 2 or Tier 3 implementation should document them as part of administration protocols.

## 7 Conclusions

Although individual variability is evident, both trained and untrained populations show altitude-related decrements in aerobic performance, while brief, isolated, anaerobic-dominant efforts are generally maintained within moderate altitude (~1,750–3,000 m). Because PES evaluations must be scientifically grounded and comparable across locations, altitude-adjusted minimum scoring rules should be considered when aerobic components can evidently influence employment outcomes. Programs can preserve comparability by (i) holding administration conditions constant across sites, (ii) applying altitude-adjusted scoring equivalents (as operationalized in the Framework section) after validation, or (iii) providing accommodations such as a defined acclimatization window, consistent with each jurisdiction’s duty to accommodate (Parliament of Australia, 1992; Supreme Court of Canada, 1999; Parliament of the United Kingdom, 2010).

Most PES evaluations blend endurance, strength, power, and direct task simulations, so they rarely map cleanly onto single fitness categories. As with other environmental stressors such as heat, testing conditions often differ from operating conditions, and environmental heat can meaningfully alter performance and risk, reinforcing the need to align administration with environment (Cheung et al., 2016). Where feasible, defined acclimatization timeframes or administration choices that minimize testing site effects help preserve fairness. At moderate altitude, Δt is small toward the lower end and increases toward the upper end, and Δt scales with task duration, so mid- to longer-duration aerobic components warrant the most attention. Consistent with the CP/W′ mechanism and our simulations, longer inter-component recovery (≥5 min passive) limits altitude-related changes in intermittent elements, while continuous or near-continuous aerobic elements are more affected.

This theoretical framework is a starting point to encourage empirical research to elucidate the effects of altitude on PES performance. If a hypoxic environment may unfairly affect one's PES evaluation performance, altitude-specific equivalent cut-scores/standards offer a practical adjustment that keeps pass/fail rates comparable across testing locations. Because administration conditions affect outcomes, test specifications should explicitly define allowable environmental ranges, including altitude, and explicitly state how scores will be interpreted or adjusted when testing occurs outside those ranges (e.g., approved altitude-equivalent cut-scores or a defined acclimatization requirement), with a plan for periodic re-validation when conditions, populations, or job demands significantly change. While this was the focus of the paper for simplicity, such a validated framework would also be useful for readiness monitoring. As mentioned previously, occupational tasks performed at altitude may require PES evaluation scoring adjusted to that altitude to improve validity. The real-world examples referenced in the Introduction were within the context of the military, but this could be applied to firefighting, emergency medical services, or search and rescue, among others. Looking ahead, scoring rules grounded in task features (duration, intensity relative to CP, work–recovery structure) and calibrated against prospective field data should reduce false negatives at altitude without inflating false positives at sea level. As a Hypothesis and Theory paper, our contribution is a set of explicit, testable predictions plus a validation roadmap; the next step is to challenge, refine, and calibrate those predictions. The framework presented here could be refined as field tests reveal its prediction ability or lack thereof. A potential long-term direction would be the development of a model or algorithm that could be applied to any PES evaluation to generate an altitude-adjusted minimum standard based on task features and work—rest structure. This would only be feasible if, after appropriate field testing, sufficient empirical evidence underlies the model such that it could withstand legal scrutiny.

## Data Availability

The original contributions presented in the study are included in the article/supplementary material. Further inquiries can be directed to the corresponding author.
